# Lignin-Based Polyols with Controlled Microstructure by Cationic Ring Opening Polymerization

**DOI:** 10.3390/polym13040651

**Published:** 2021-02-22

**Authors:** Jonatan Perez-Arce, Ander Centeno-Pedrazo, Jalel Labidi, Jose R. Ochoa-Gomez, Eduardo J. Garcia-Suarez

**Affiliations:** 1 TECNALIA, Basque Research and Technology Alliance (BRTA), Alava Technology Park, Leonardo da Vinci 11, 01510 Vitoria-Gasteiz, Spain; jonatan.perez@tecnalia.com (J.P.-A.); ander.centeno@tecnalia.com (A.C.-P.); 2 Biorefinery Processes Research Group, Department of Chemical and Environmental Engineering, University of Basque Country UPV/EHU, Plaza Europa 1, 20018 Donostia-San Sebastián, Spain; jalel.labidi@ehu.eus; 3 Center for Cooperative Research on Alternative Energies (CIC energiGUNE), Basque Research and Technology Alliance (BRTA), Alava Technology Park, Albert Einstein 48, 01510 Vitoria-Gasteiz, Spain; 4 IKERBASQUE, Basque Foundation for Science, Maria Diaz de Haro 3, 48013 Bilbao, Spain

**Keywords:** lignin upgrading, lignin-based polyols, controlled microstructure, biomass valorization

## Abstract

Lignin-based polyols (LBPs) with controlled microstructure were obtained by cationic ring opening polymerization (CROP) of oxiranes in an organosolv lignin (OL) tetrahydrofuran (THF) solution. The control on the microstructure and consequently on the properties of the LBPs such as hydroxyl number, average molecular weight, melting, crystallization and decomposition temperatures, are crucial to determine the performance and application of the derived-products. The influence of key parameters, for example, molar ratio between the oxirane and the hydroxyl groups content in OLO, initial OL concentration in THF, temperature, specific flow rate and oxirane nature has been investigated. LBPs with hydroxyl numbers from 35 to 217 mg KOH/g, apparent average M_w_ between 5517 and 52,900 g/mol and melting temperatures from −8.4 to 18.4 °C were obtained. The CROP procedure allows obtaining of tailor-made LBPs for specific applications in a very simple way, opening the way to introduce LBPs as a solid alternative to substitute currently used fossil-based polyols.

## 1. Introduction

Diminishing fossil fuel reserves and degradation of the environment are highly important concerns nowadays. These concerns push the scientific community to look for new and efficient processes to convert renewable biomass feedstock, mainly lignocellulosic biomass, into both high value chemicals and liquid fuels [1]. Thus, the production of added-value biochemicals and biomaterials by biorefinery approaches using non-edible bioresources is in high demand [2]. However, it still remains a big challenge for bio-based chemicals to compete with petroleum-based ones in price, performance and quality of the final products. New efficient and economically attractive routes for the preparation of bio-based materials able to compete with their fossil-based counterparts are needed [3,4,5,6,7].

In this context, polyols are chemicals with a huge market (approximately USD 26.2 billion/y in 2019 and a forecasting of USD 34.4 billion for 2024) and a broad spectrum of applications, for example, polymers, energy storage, et cetera, but currently mainly obtained from petroleum [7]. Therefore, polyols are good targets to be obtained from lignocellulosic biomass. Lignin, which is obtained in huge amounts as by-products in the pulp industry [8,9], is probably one of the most interesting and abundant bioresources for the synthesis of industrially useful bio-based polyols [10,11]. In this sense, our research group has recently reported a new, greener and economic route to obtain lignin-based polyols (LBPs) under very mild reaction conditions by cationic ring opening polymerization (CROP), substantially improving the more traditional routes to obtain LBPs such as either lignin fragmentation [12,13,14], lignin oxypropylation, or both, in basic media [15,16], which are carried out under harsher reaction conditions [17,18]. On the other hand, the total control of polyol properties such as hydroxyl number (number of –OH per gram of polyol), average molecular weight, thermal behavior (melting, crystallization, decomposition temperatures), linear or branched and viscosity, are crucial since they will determine the performance and the best application of the final derived products, for example, foams, adhesives, coatings, energy storage materials, et cetera.

In this work, we reported the synthesis of LBPs by CROP of oxiranes in an organosolv lignin (OL) THF solution and the investigation on how key reaction parameters such as, oxirane/–OH-lignin-groups (oxirane/OH–OL) molar ratio, initial OL concentration, temperature, oxirane specific addition flow rate (Q_s_) and oxirane nature influence the physicochemical properties of the obtained LBPs.

## 2. Experimental

### 2.1. Materials

Propylene oxide (PO, 99.5%), butylene oxide (BO, 99%), epichlorohydrin (oxiranes, 99%), glycidol (GLY, 96%), 1,2-epoxyhexane (HO, 97%), 1,2-epoxyoctane (OO, 96%), 1,2-epoxy-5-hexene (HEO, 98%), boron trifluoride etherate (BF_3_, 48%) and tetrabutylammonium hydroxide (0.1 *N* in toluene/methanol) were purchased from Acros Organics (Geel, Belgium). *N*,*N*-dimethylethylamine (99%) was purchased from Sigma-Aldrich (San Luis, MO, USA). Tetrahydrofuran (THF, 99%) and acetonitrile (ACN, 99%) were purchased form Fisher (Waltham, MA, USA). The OL was kindly supplied by TNO (Netherlands Organisation for Applied Scientific Research, The Hague, The Netherlands) and it was a lignin mixture from various wood types containing 1.18 mmol/g of OH-aliphatic and 3.33 mmol/g of OH-aromatics. OL composition is 98.16 wt.% (91.64 wt.% of Klason lignin and 6.52 wt.% of acid soluble lignin), with cellulose, hemicellulose and ash contents around 0.01 wt.%, 0.35 wt.% and 0.40 wt.%, respectively.

### 2.2. Synthesis of LBPs

A typical synthesis was carried out in a 125 mL four-necked glass jacketed reactor equipped with a mechanical stirrer at the desired temperature and at atmospheric pressure. Previously, the OL was dried at 50 °C in a vacuum oven (under 20 mbar pressure) for 24 h. Then, a solution of the OL in THF was placed into the reactor, and the desired amount of catalyst (boron trifluoride etherate) was added under stirring. Next, the stablished amount of the selected oxirane was added continuously using a syringe pump at a chosen Q_s_ (defined as mL of oxirane per OL hydroxyl groups per hour). Once oxirane addition was completed the reaction was kept under stirring for an additional 1 h of post-addition time to ensure its total conversion. Finally, the reaction was quenched with an excess of *N*,*N*-dimethylethylamine (1.66 equivalents referred to the catalyst) and the solvent was removed in a rotary evaporator until constant weight yielding the desired LBP, generally, as a dark brown viscous liquid. All LBPs were dried at 65 °C under vacuum (10 mbar) overnight prior to analyses.

### 2.3. Characterization of LBPs

Attenuated total reflection Fourier transform infrared (ATR-FTIR) spectroscopy analyses were performed using an infrared spectrophotometer Bruker Instrument (Billerica, MA, USA) model ALPHA-P in the range of 4000 to 400 cm^−1^ at a resolution of 4 cm^−1^.

Molecular mass distributions (MWD), the apparent weight average molecular masses (M_w_) and the dispersity indexes (DI) were determined by size exclusion chromatography (SEC) using a Knauer Azura liquid chromatography equipment (Berlin, Germany). The system consisted of two columns (Agilent ResiPore, Santa Clara, CA, USA, 7.5 × 300 mm, 3 µm) and a pre-column connected in series and an Knauer Azura RID 2.1L refractive index detector (Berlin, Germany). Dried THF at a flow rate of 1 mL/min at 40 °C was the mobile phase. Calibration was carried out with an Agilent (Santa Clara, CA, USA) polymer polystyrene standard kit consisting of 15 standards with Mw ranging from 162 to 278,700 g/mol.

The OL and LBPs hydroxyl numbers (OH#) were determined according to American Society for Testing and Materials (ASTM) E-1899-02 standard using THF as a solvent in a 702 SM Titrino equipment from Metrohm (Herisau, Swizerland).

Differential scanning calorimetry (DSC) analyses were carried out in a TA Instruments Q1000 modulated differential scanning calorimeter (New Castle, UK). Scans cycles consisted of heating the sample up to 100 °C followed by a cooling ramp between 100 and −80 °C and a subsequent heating ramp between −80 and 220 °C, both at 10 °C/min.

Thermo-gravimetric analysis (TGA) of LBPs was performed on a TA instruments TG-DTA92 instrument (New Castle, UK) under air atmosphere. In a typical experiment, samples were heated from 20 to 100 °C at 5 °C/min. Then, samples were dried at this temperature for 1 h in order to eliminate moisture and residual solvent, if any, and finally samples were heated from 120 to 700 °C at 10 °C/min while recording the mass losses.

Oxirane conversions were calculated by subtracting the unreacted oxirane amount from the total added during the reaction, and dividing the result by the total oxirane amount added. The unreacted oxirane was determined by HPLC instrument (Agilent 1260 Infinity, Santa Clara, CA, USA) fitted with a 300 mm × 7.8 mm × 9 µm Aminex HPX-87 column and a refractive index detector. The mobile phase was 0.01 N aqueous sulfuric acid and the flow rate 0.7 mL/min. Column and detector temperatures were 65 °C and 50 °C, respectively.

### 2.4. LBP Composition Determination

There are three components in the final LBPs: OL, employed oxirane and the copolymerized solvent (THF). Previously, to determine the composition of the LBP, excess solvent was removed until a constant weight of the LBP was obtained. The OL wt.% in the LBP was calculated by dividing the initial mass of OL (mL) employed in the reaction by the total weight of the LBP obtained (mLBP) and multiplying the result by 100. The oxirane weight content in the LBP (oxirane wt.%) was calculated by dividing the difference between the oxirane weight (m_oxirane_) used in the reaction and the unreacted oxirane mass by the total weight of LBP (mLBP) obtained and multiplying the result by 100. Copolymerized THF contents (THF wt.%) were calculated by subtracting the sum of the two above amounts from 100.

## 3. Results and Discussion

### Synthesis of the LPBs Family with Controlled Microstructure by CROP

**Scheme 1:**
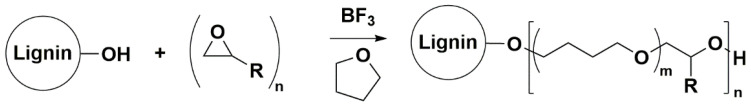
Lignin-based polyols (LBPs) synthesis by cationic ring opening polymerization (CROP) reaction of oxiranes in THF in the presence of lignin.

LBPs were synthesized by CROP of oxiranes and THF in the presence of a OL source using a Lewis acid catalyst (BF_3_·OEt_2_) (Scheme 1) being both the THF comonomer and solvent as previously discussed.

Based on our previous work [17], the reaction parameters that showed little or negligible influence in the LBP microstructure, namely, the catalyst amount and the post-addition were selected. Therefore, the catalyst amount and the post-addition time were set at 0.125 catalyst/–OH–OL molar ratio and 1 h, respectively. Under the selected conditions, reaction parameters such as the oxirane/OH–OL molar ratio, the OL initial concentration, the reaction temperature, the specific flow rate of oxirane addition and the nature of the oxirane were studied to understand how they influence the LBPs’ microstructures using butylene oxide as the oxirane. However, before discussing the influence of these parameters, it is necessary to understand the propagation and termination step mechanisms of the CROP reaction in the presence of THF using lignin as the polyol initiator.

**Scheme 2:**
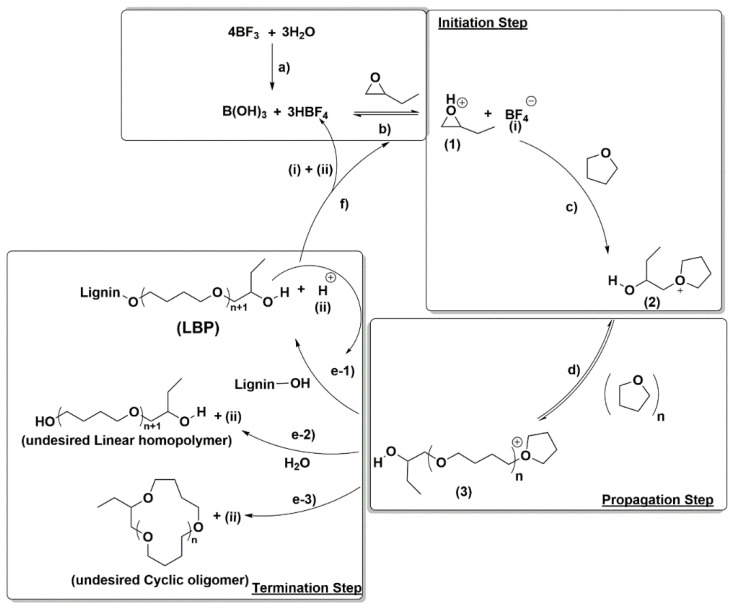
Proposed CROP polymerization with butylene oxide as oxirane in THF in the presence of lignin adapted from our previous work [17].

As depicted in Scheme 2, after the initiation, the propagation step takes place by active chain end (ACE) mechanism by reaction of the formed cation 2 with THF leading to the growing polymer (3). This propagation step might also occur by reaction of cations 2 and 3 with the oxirane, but this is unlikely because THF is used as reaction media and therefore it is in a large excess relative to the oxirane due to the slow semicontinuous oxirane addition to the reaction mixture. On the other hand, the termination could potentially occur in three different ways: (1) by reaction of cation 3 with the –OH groups present in the lignin leading to the desired LBP or with an already formed LBP (path e-1); (2) by reaction with traces of water leading to an undesired linear homopolymer (path e-2); and (3) by back-biting reaction between the terminal –OH moiety in cations 3 with a carbon atom in the same cation leading to an undesired cyclic oligomer (COL) (path e-3). Consequently, in view of the CROP mechanism at the selected reaction conditions the competition between homopropagation (path d) and termination reactions (paths e-1, e-2 and e-3) will depend mainly on the THF and –OH concentrations as already reported in the literature [19,20]. Even if both reaction mechanisms, activated monomer (AM) and ACE mechanisms, occur simultaneously, one is preferred to the other depending on THF and –OH concentrations, allowing playing with those parameters to fine tune the final LBPs microstructure for a required application. Another variable playing a key role in the LBP microstructure is the reaction temperature; the higher temperature the lower THF incorporation in the final LBP [21,22]. On the basis of previously discussed premises, the influence of oxirane/–OH–OL molar ratio (affecting THF concentration), the influence of the lignin concentration (affecting the THF concentration and the –OH concentration), the effect of the temperature (affecting the THF incorporation), the influence of the oxirane specific addition flow rate (Q_s_) (affecting the instant concentration-consumption of the oxirane) and once all these parameters were studied with the BO the influence of the oxirane nature (affecting mainly the thermal properties of the LBPs) was investigated as well.

- Effect of the oxirane/–OH–OL molar ratio on the LBPs microstructure

This parameter was studied for values ranging from 1 to 5 using butylene oxide (BO) as an oxirane. As already stated, it is clear that the oxirane monomer acts as promotor in the CROP reaction and then it is expected that LBPs having higher molecular weights could be obtained by increasing the oxirane/OH–OL molar ratio. Experimental conditions of reactions carried out and results obtained are given in Table 1 together with the characteristics of the starting OL (entry 1). The BO content in LBPs increases with BO/OH–OL molar ratio from 8 wt.% at a molar ratio of 1 to 16.7 wt.% at a molar ratio of 5. As expected, the OL content in the final LBP decreases from 24.5 wt.% at a BO/OH–OL molar ratio of 1 (entry 2) to 10.3 wt.% at a BO/OH–OL molar ratio of 5 (entry 3). Concerning THF incorporation into the final LBPs, it increases slightly with molar ratio. These results can be explained on the basis of the proposed CROP reaction mechanism since by increasing the oxirane content and consequently the relative ratio between oxirane/–OH–OL the propagation step is favored versus the termination one. On the other hand, the LBP hydroxyl number decreases by increasing the molar ratio from 81 to 35 mg KOH/g due to the increased amount of BO incorporated into the LBP because molecular mass increases with BO incorporation but OH groups introduced into a growing chain are independent of the number of BO units incorporated. The main difference between the three prepared LBPs at different molar ratios was observed in their crystallization and the melting temperatures, the lower the molar ratio the higher the crystallization temperature as a consequence of the lower length of flexible polyether chains. On the other hand, the melting temperature decreases when the molar ratio increases which can be attributed to the longer polyether moieties in the final LBP leading to a more flexible structure [23].

The hydroxyl numbers (OH#), are by far lower than the OH# of the employed OL (243 mg KOH/g) (entry 1, Table 1) versus 81 mg KOH/g for a molar ratio of 1 (entry 2, Table 1), 70 mg KOH/g for a molar ratio of 2 (entry 3, Table 1) and 35 mg KOH/g for a molar ratio of 5 (entry 4, Table 1) since the apparent average M_w_ of the final LBPs increased considerably with the molar ratio, M_w_ 989 g/mol being the apparent average for OL (entry 1, Table 1) and 7172 g/mol, 11,263 g/mol and 30,855 g/mol for LBPs obtained with BO/OH–OL molar ratios of 1, 2 and 5, respectively (entries 2–4, Table 1).

**Figure 1:**
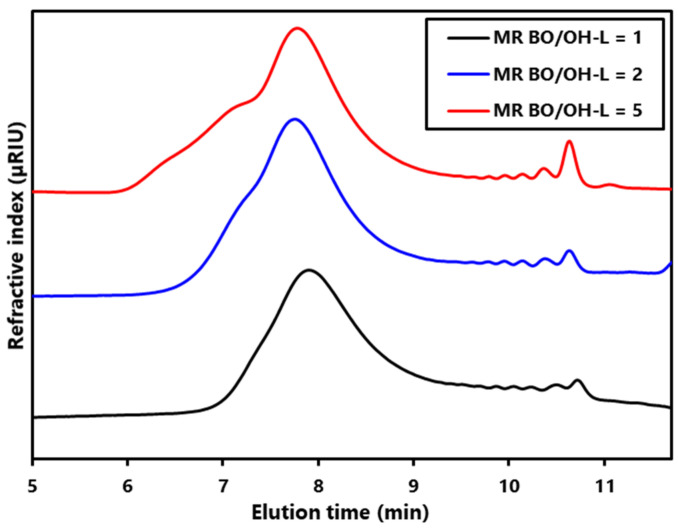
Influence of the BO/OH-OL molar ratio on the LBPs M_w_D.

The LPBs were analyzed by ATR-FTIR as well, but no significant differences between them were found as it can be seen in supporting information (Figure S2). As expected, the MWD of the LBPs increased with the amount of BO incorporated (Figure 1) [24].

However, at long elution times, it can be observed that the small peaks attributed to cyclic oligomers (COLs) formation increase with molar ratio, as reported in the literature [20]. In fact, it has been reported for the copolymerization of THF and ethylene oxide that the formation of COLs is lower or suppressed at THF conversion below 60% [19]. As it can be seen in Table 1, THF content in LBPs, and hence THF conversion, increases with molar ratio so that the lower the BO/OH–OL molar ratio the lower the amount of COL formed.

Concerning the decomposition temperature, it is observed that the LBPs have two degradation main peaks; one with the maximum weight lost ranging from ca. 360 to ca. 380 °C and another one with a maximum weight lost at ca. 540 °C (see Figures S1 and S3). Relative to the decomposition of the initial OL, two decomposition peaks can be observed, the first one at 385 °C and the second and more intense one at around 530 °C (See Figure S1). Therefore, the first weight lost in the LBP can be attributed to the polyether chain grafted to the OL core and the second one to the decomposition of the core OL. It seems clear that the thermal degradation pattern of the obtained LBPs at different molar ratios is very similar and it could be concluded that within the LBP composition range obtained the amount of added oxirane has no significant impact on the final LBPs thermal degradation; these results being consistent with those obtained in the copolymerization of styrene oxide with propylene oxide using diols as initiators [25].

- Effect of the OL initial concentration on the LBPs microstructure

The OL concentration plays a crucial role in the polymerization process because OL concentration is directly related to the –OH groups available in the reaction media. The competition between the –OH groups and THF concentration is key in the CROP reaction because the prevalence of one of them has significant influence in the propagation and termination steps. In fact, the THF homopropagation competes with the termination by OL–OH groups as shown in Scheme 2. Consequently, the OL initial concentration does affect the microstructure of the final LBPs allowing tuning of the final LBP properties. Several CROPs at four different initial OL concentrations (55 g/L, 110 g/L, 164 g/L and 274 g/L) at two different BO/OH–OL molar ratios (1 and 2) were carried out. Experimental conditions and results are given in Table 2. A clear trend is observed at the two BO/OH–OL molar ratios studied, in other words, the lower the OL initial concentration the higher the apparent average M_w_ of the obtained LBP, which makes sense since by increasing the initial OL concentration there is an increase in the –OH concentration increasing the probability of the termination step by attack of the –OH to the growing polymer (Scheme 2) avoiding the THF incorporation and consequently obtaining LBPs with lower apparent average Mw. Looking at the OH#, the lower the OH# the higher the apparent average M_w_ of the LBP obtained. The OH# is a crucial parameter for the polyol’s application scope determining, in most cases, their final application. In this sense, as it can be seen in Table 2, different LBPs can be obtained with OH# ranging from 39 (entry 5, Table 2) to 127 mg KOH/g (entry 4, Table 2) by only tuning either the molar ratio, initial OL concentration, or both. The crystallization and melting temperatures of the resulting LBPs are also related to the OH# number and the apparent average M_w_ being both lower when increasing the OH#. It was found that there is not significant difference in the trend followed by the resulting LBPs at molar ratios 1 and 2 (entries 1–4 and entries 5–8, Table 2). With initial OL concentrations of 55 g/L, 110 g/L and 164 g/L the main difference between the obtained LBPs at BO/OH–OL molar ratio of 1 or 2 is the apparent average M_w_ of the final LBPs, having those obtained at molar ratio 1 generally having a lower apparent average M_w_ (entries 1 and 5, entries 2 and 6 and entries 3 and 7, Table 2). The lower apparent average M_w_ of the LBPs obtained at the lower molar ratio is attributed to the lower amount of oxirane used. Nevertheless, when the initial OL concentration is 274 g/mL almost no difference in the obtained LBPs at both studied molar ratios is observed (entries 4 and 8, Table 2). This fact could be attributed to the large concentration of OL leading to a high amount of –OH groups ready to attack the growing polymer, thus predominating the termination step over the propagation one.

The ATR-FTIR spectra (see Figure S4) of the obtained LBPs at the initial OL concentrations tested at molar ratios 1 and 2 were similar and no appreciable differences were found. The only difference is just the more intense –OH stretching band at around 3500 cm^−1^ related to the intensity of the –OH group band in the LBPs with higher OL content.

Concerning the thermal degradation of obtained LBPs as a function of initial OL concentration, at both molar ratios it follows a similar trend as observed by TGA (see Figure S5). In all cases, the decomposition temperature of the LBPs is in the range from 350 to 365 °C showing those obtained at a lower BO/OH–OL molar ratio’s slightly higher decomposition temperatures as observed before. Another weight loss between 490 and 530 °C is observed for all LBPs, attributed to the OL core whose maximum weight lost temperature is ca. 530 °C (see Figure S1).

- Effect of the reaction temperature on the LBPs microstructure

From a kinetic point of view, looking into the Arrhenius equation the rate constant (k) in a reaction increases with the temperature and this is the case of the CROP reaction with 3-member cyclic ethers (oxiranes) where the polymerization reaction is irreversible [26]. Nevertheless, in the case of four or more-member cyclic ethers, such as THF, even if the polymerization rate is faster by increasing the CROP temperature, the equilibrium monomer concentration is higher and consequently the monomer conversion lower, thus obtaining higher molecular weight polymers at low temperatures [22].

Consequently, if THF is employed as the solvent, it should be taken into account that its homopropagation is closely related to the reaction temperature. The higher the temperature, the higher the monomer equilibrium concentration and consequently less THF will be able to polymerize and will be incorporated into the final polymer [20]. Therefore, the temperature could be used to control the THF homopolymerization, its incorporation into the final LBP and, as a consequence, the LBP final properties.

The influence on LBP microstructure of three reaction temperatures were investigated, 5 °C, 25 °C and 55 °C. Experimental conditions and results are given in Table 3. As expected, the higher the temperature the lower the THF incorporation in the LBP, 75.3 wt.%, 71.5 wt.% and 59 wt.% at 5°C, 25 °C and 55 °C, respectively. The apparent average M_w_ of the obtained LBPs decreases drastically when temperature increases. Concerning the crystallization and melting temperatures, the LBPs with less THF incorporated, those obtained at higher temperatures, show both lower crystallization and melting temperatures. As in the case of the other studied variables, the ATR-FTIR spectra of the obtained LBPs varying the temperature do not show significant changes (Figure S6). The decomposition temperature showed by TGA is quite similar in the three LBPs with a maximum weight lost at ca. 350–365 °C and another weight loss at around 440 °C attributed to the core OL decomposition, with this peak being more pronounced in the LBPs with higher OL content (Figure S7), as expected.

- Influence of BO specific addition flow rate (Q_s_) on the LBPs microstructure

The oxirane specific addition flow rate (Q_s_) has a strong influence on the instant oxirane concentration in the reaction medium. Consequently, the formation of the undesired COLs could be avoided depending on how fast the oxirane is incorporated into the growing chain. The incorporation of the oxirane as well as its reactivity will then depend on the Q_s_ but also on the oxirane nature. To evidence the importance of the Q_s_ on the obtention of the final wanted LBP this study was done using BO as oxirane. Three different Q_s_ were tested (36.5, 73.0 and 146.0 mL/OH–OL/h). CROP experimental conditions and results obtained are given in Table 4. As can be seen, there are no major differences in terms of OL, BO and THF contents in the resulting LBPs and, consequently, there are also no significant differences in their properties.

Along the same lines, the ATR-FTIR spectra and the thermal degradation analysis (TGA) profiles of the LBPs do not show any significant difference between them as constated in supporting information, Figures S8 and S9, respectively.

- Effect of the oxirane nature on the LBPs microstructure

**Figure 2:**
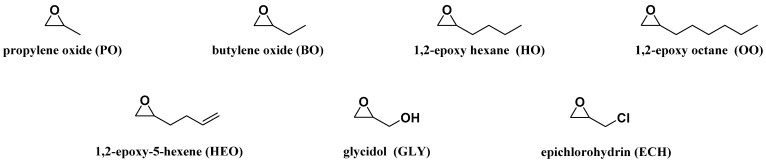
Oxiranes selected to study their influence in the LPBs microstructure.

Several oxiranes depicted in Figure 2 were selected to investigate the influence on the LBPs microstructure of: (1) the length of the oxirane alkyl chain, PO (1 carbon atom), BO (2 carbon atoms), HO (4 carbon atoms) and OO (6 carbon atoms); and (2) the presence of a functional group pendant on the CH_3_ carbon of the PO: an allylic moiety (HEO), a hydroxyl group (GLY) and a chlorine group (oxiranes). The influence of the oxirane nature on the LBP properties, namely, OH#, melting and crystallization temperatures and the apparent average M_w_, is shown in Table 5.

The aliphatic chain length was found to influence the THF monomer incorporation into the final LBP. Both the THF and OL contents on the final LBP decrease with the increase of the alkyl chain length while the oxirane content in the LBP increases with the length of the alkyl chain (entries 1–4, Table 5). On the other hand, the crystallization temperature of the obtained LBPs decreases with the increase in aliphatic chain length up to 4 carbons while melting temperatures increase with increasing the number of carbons up to 4 into the aliphatic chain of the oxirane (entries 1–2, Table 5). When the number of carbons in the aliphatic chain of the oxirane is 4 or 6, HO and OO, respectively, there was no significant difference found between the crystallization or melting temperatures (entries 3 and 4, Table 5) [23]. The presence in the oxirane of a –OH pendant group, as in the case of GLY, was found to strongly influence the melting temperature, 7.7 °C, (entry 6, Table 5) compared to the other LBPs. The presence of a pendant double bond (HEO) or a –Cl group (oxiranes) leads to similar crystallization and melting temperatures (entries 5 and 7, Table 5) but their melting temperatures (14.4 °C and 14.1 °C) are higher among the obtained LBPs, probably due to polar-polar interactions between polyether chains. Concerning the OH#, the LBPs obtained with the employed oxiranes show lower OH#, in the range from 58 to 79 mg KOH/g, than the starting OL as expected, except for GLY showing the corresponding LBP a OH# similar to the OL due to the presence of an extra –OH group in the LBP per GLY unit incorporated.

The presence of a double bond in oxirane HEO compared with oxirane HO (entries 3 and 5, Table 5) clearly affects the composition of the final obtained LBP, in the presence of the double bond the content of THF increases from 72.3 to 78.8 wt.% while both OL and oxirane contents decrease in the LBP containing HEO. This fact could be explained because the double bond is withdrawing electron density on the carbon in two positions of the oxirane facilitating the THF incorporation. A similar behavior was observed with the oxiranes, an oxirane with another pendant electron-withdrawing group (–Cl). The apparent average M_w_ distributions of the prepared LBPs show similar molecular patterns, a main peak of higher apparent average M_w_ than that of the starting OL with a series of small peaks of lower apparent average M_w_ attributed to the formation of COLs (see Figure S10). The LBPs with the highest apparent average M_w_ were obtained with HEO and oxiranes as the oxiranes, 52,900 and 35,594 g/mol, respectively, due to a higher THF incorporation into the LBP attributed to the presence of electron-withdrawing pending functionalities (entries 5 and 7, Table 5). On the contrary, the LBP with the lowest apparent average M_w_ was obtained with GLY, 9373 g/mol, due to the lower THF incorporation into the final LBP (entry 6, Table 5).

The ATR-FTIR spectra of these LBPs follow the same pattern with no significant variations but those attributed to stretching bands of C–Cl for oxiranes at 745 cm^−1^ and of C=C for HEO at 910 cm^−1^ and a more intense –OH stretching band for GLY (see Figure S11). The thermal stability of the LBPs was followed by TGA and all LBPs have similar degradation profiles with two main mass loss peaks, the first one at ca. 350–380 °C attributed to the polyether chains grafted to the OL core and the second one at ca. 530 °C attributed to the degradation of the OL core (Figure S12).

## 4. Conclusions

It has been demonstrated that families of lignin-based polyols with different thermal, physical and chemical properties can be obtained by CROP reaction by fine tuning key reaction parameters such as the oxirane/OH-lignin groups’ molar ratio, the OL initial concentration, the temperature, the specific flow rate of oxirane addition and the nature of the oxirane. The total control of the studied reaction variables allowed obtaining a plethora of LBPs with very well-defined properties, for example, hydroxyl number, apparent average M_w_ as well as crystallization and melting temperatures on demand for a specific application. This control of the microstructure of the resulting LBPs and their properties is carried out in a very simple way at very mild reaction conditions. This procedure and the total LBPs’ properties control will definitively pave the way to introduce LBPs as a solid alternative to completely or partially substituting currently used fossil-based polyols in several industrial applications.

## Figures and Tables

**Table 1:** Influence of the BO amount on the LBP microstructure ^a^.

Entry	BO/OH-OL ^b^ (mol)	OL (wt.%) ^c^	BO (wt.%) ^c^	THF (wt.%) ^c^	Tc (°C)	Tm (°C)	OH# (mg KOH/g)	Mw ^d^ (g/mol)	D ^e^ (M_w_/M_n_)
1 ^f^	0	98.16	0	0	-	-	243	989	2.1
2	1	24.5	8.0	67.5	−17.3	11.5	81	7172	2.7
3	2	19.4	12.6	68	−20.8	9.7	70	11,941	3.6
4	5	10.3	16.7	73	−33.8	3.7	35	30,855	6.9

^a^ Reaction conditions: temperature = 25 °C; pressure = atmospheric; catalyst/lignin–OH molar ratio = 0.125; OL concentration (g/L) = 110; specific flow rate (oxirane) = 73 mL·mol–OH(OL)^−1^·h^−1^; post-addition time = 1 h; ^b^ butylene oxide/lignin–OH groups molar ratio molar ratio; ^c^ content in the LBP; ^d^ average apparent M_w_; ^e^ dispersity index. ^f^ starting OL.

**Table 2:** Influence of the OL concentration on the LBP microstructure ^a^.

Entry	OL (g/mL)	BO/OH–OL ^b^ (mol)	OL (wt.%) ^c^	BO (wt.%) ^c^	THF (wt.%) ^c^	Tc (°C)	Tm (°C)	OH# (mg KOH/g)	Mw ^d^ (g/mol)	D ^e^ (M_w_/M_n_)
D1	55	1	15.7	5.1	79.2	−8.1	18.4	60	14,224	3.1
2	110	1	24.5	8.0	67.5	−17.3	11.5	81	8172	2.7
3	164	1	28.6	9.3	62.1	−29.6	10.2	103	7282	2.4
4	274	1	34.2	11.1	54.7	n.f. ^f^	3.6	127	7512	2.6
5	55	2	13.8	9.0	77.2	−16.0	16.4	39	27,272	5.0
6	110	2	17.3	11.2	71.5	−20.3	9.7	56	12,534	3.5
7	164	2	24.9	16.2	58.8	−27.3	5.0	83	9669	3.1
8	274	2	27.4	17.8	54.8	n.f. ^f^	−8.4	117	6921	2.8

^a^ Reaction conditions: temperature = 25 °C; pressure = atmospheric; catalyst/OL–OH molar ratio = 0.125; oxirane specific addition flow rate = 73 mL·mol–OH(OL)^−1^·h^−1^; post-addition time = 1 h; ^b^ butylene oxide/lignin–OH groups molar ratio; ^c^ content in the LBP; ^d^ apparent average M_w_; ^e^ dispersion index; ^f^ n.f. = not found.

**Table 3:** Influence of the temperature on the LBP microstructure ^a^.

Entry	Temperature (°C)	OL (wt.%) ^b^	BO (wt.%) ^b^	THF (wt.%) ^b^	Tc (°C)	Tm (°C)	OH# (mg KOH/g)	Mw ^c^ (g/mol)	D ^d^ (M_w_/M_n_)
1	5	15	9.7	75.3	−18.9	12.5	42.5	37,754	7.0
2	25	17.3	11.2	71.5	−20.2	9.7	64.5	11,263	3.6
3	55	24.9	16.2	59	−27.8	−6.3	107.1	5517	2.2

^a^ Reaction conditions: molar ratio BO/OH–OL = 2:1; pressure = atmospheric; catalyst/lignin –OH molar ratio = 0.125; OL concentration (g/L) = 110; specific flow rate (oxirane) = 73 mL·mol–OH(lignin)^−1^·h^−1^; post-addition time = 1 h; BO/OH–OL (mol) = 2; ^b^ content in the LBP; ^c^ apparent average M_w_; ^d^ dispersity index.

**Table 4:** Effect of the oxirane specific addition flow rate (Q_s_) on the LBP microstructure ^a^.

Entry	Q_s_ (mL/nOH–OL/h)	OL (wt.%) ^b^	BO (wt.%) ^b^	THF (wt.%) ^b^	Tc (°C)	Tm (°C)	OH# (mg KOH/g)	Mw ^c^ (g/mol)	D ^d^ (M_w_/M_n_)
1	36.5	16.0	10.4	73.6	−25.8	12.1	79	14,535	3.7
2	73.0	16.6	10.8	72.6	−20.3	9.7	76	12,534	3.5
3	146.0	15.8	10.2	74.0	−24.5	12.7	73	19,732	5.1

^a^ Reaction conditions: temperature = 25 °C; pressure = atmospheric; catalyst/lignin–OH molar ratio = 0.125; OL concentration (g/L) = 110; BO/OH–OL (mol) = 2; post-addition time = 1 h; ^b^ content in the final LBP; ^c^ apparent average M_w_; ^d^ dispersity index.

**Table 5:** Influence of the oxirane nature on the LBP microstructure ^a^.

Entry	Oxirane	OL (wt.%) ^b^	Oxirane (wt.%) ^b^	THF (wt.%) ^b^	Tc (°C)	Tm (°C)	OH# (mg KOH/g)	Mw ^c^ (g/mol)	D ^d^ (M_w_/M_n_)
1	PO	16.5	8.7	74.8	−24.9	9.9	77	15,090	4.0
2	BO	16.0	10.4	73.6	−25.8	12.1	79	14,535	3.7
3	HO	14.5	13.1	72.3	−32.2	11.6	58.1	25,747	5.5
4	OO	14.4	16.5	69.1	−31.9	11.2	57.4	26,007	5.4
5	HEO	11.2	9.5	78.8	−27.6	14.4	50	52,900	11.8
6	GLY	20.3	13.5	66.2	n.f. ^e^	7.7	217	9373	3.4
7	ECH	12.0	10.0	78.0	−28.2	14.1	63	35,594	7.3

^a^ Reaction conditions: temperature = 25 °C; pressure = atmospheric; catalyst/OL–OH molar ratio = 0.125; specific addition flow rate (Q_s_) (oxirane) = 36.5 mL·mol–OH(OL)^−1^·h^−1^; post-addition time = 1 h; ^b^ content in the LBPs; ^c^ apparent average M_w_; ^d^ dispersity index; ^e^ n.f. = not found.

## Supplementary Materials

The following are available online at https://www.mdpi.com/2073-4360/13/4/651/s1, Figure S1: (a) TGA profile of the OL, (b) first derivative of the TGA of the OL, Figure S2: ATR-FTIR spectra of the LBPs obtained at different BO/OH-L molar ratios, Figure S3: (a) TGA profiles of the LBPs obtained at different BO/OH-L molar ratios; (b) first derivatives of the TGA at different BO/OH-L ratios, Figure S4: ATR-FTIR spectra of the LBPs obtained at different OL concentrations: (a) BO/OH-L molar ratio 1 and (b) BO/OH-L molar ratio 2, Figure S5: (a) TGA profiles of the LBPs obtained at different OL concentrations with BO/OH-L molar ratio 1; (b) first derivative of the TGA obtained at different OL concentrations with BO/OH-L molar ratio 1; (c) TGA profiles of the LBPs obtained at different OL concentrations with BO/OH-L molar ratio 2 and (d) first derivative of the TGA obtained at different OL concentrations with BO/OH-L molar ratio 2, Figure S6: ATR-FTIR spectra of the LBPs obtained at different reaction temperatures, Figure S7: (a) TGA profiles of the LBPs obtained at different reaction temperatures and (b) first derivative of the TGA at different reaction temperatures, Figure S8: ATR-FTIR spectra of the LBPs obtained at different oxirane specific addition flow rates (Q_s_), Figure S9: (a) TGA profiles of the LBPs obtained and (b) first derivative of the TGA, at different oxirane specific addition flow rates (Q_s_), Figure S10: GPC chromatograms of the obtained LBPs with different oxiranes, Figure S11: ATR-FTIR spectra of the LBPs obtained with different oxiranes, Figure S12: (a) TGA profiles of the LBPs obtained with different oxiranes and (b) first derivative of the TGA obtained with different oxiranes.

## References

[1] Alonso D.M., Wettstein S.G., Dumesic J.A. (2012). Bimetallic catalysts for upgrading of biomass to fuels and chemicals. Chem. Soc. Rev..

[2] Deutschmann R., Dekker R.F.H. (2012). From plant biomass to bio-based chemicals: Latest developments in xylan research. Biotechnol. Adv..

[3] Faruk O., Sain M. (2015). Lignin in Polymer Composites.

[4] Dragone G., Kerssemakers A.A.J., Driessen C.J.L.S.P., Yamakawa K., Brumano L.P., Mussatto S.I. (2020). Innovation and strategic orientations for the development of advanced biorefineries. Bioresour. Technol..

[5] Rinaudo M. (2006). Chitin and chitosan: Properties and application. Prog. Polym. Sci..

[6] Ioannidou S.M., Pateraki C., Ladakis D., Papapostolou H., Tsakona M., Vlysidis A., Kookos I.K., Koutinas A. (2020). Sustainable production of bio-based chemicals and polymers via integrated biomass refining and bioprocessing in a circular bioeconomy context. Bioresour. Technol..

[7] https://www.european-bioplastics.org/market.

[8] Katahira R., Elder T.J., Beckham G.T. (2018). Chapter 1. A Brief Introduction to Lignin Structure. Lignin Valorization: Emerging Approaches.

[9] Dessbesell L., Paleologou M., Leitch M., Pulkki R., Xu C. (2020). Global lignin supply overview and kraft lignin potential as an alternative for petroleum-based polymers. Renew. Sustain. Energy Rev..

[10] Gandini A. (2011). The irruption of polymers from renewable resources on the scene of macromolecular science and technology. Green Chem..

[11] Babu R.P., O’Connor K., Seeram R. (2013). Current progress on bio-based polymers and their future trends. Prog. Biomater..

[12] Schuler J., Hornung U., Kruse A., Dahmen N., Sauer J. (2017). Hydrothermal Liquefaction of Lignin. J. Biomater. Nanobiotechnol..

[13] Schuler J., Hornung U., Dahmen N., Sauer J. (2019). Lignin from bark as a resource for aromatics production by hydrothermal liquefaction. GCB Bioenerg..

[14] Sequeiros A., Serrano L., Briones R., Labidi J. (2013). Lignin liquefaction under microwave heating. J. Appl. Polym. Sci..

[15] Mozheiko L.N., Gromova M.F., Bakalo L.A., Sergeyeva V.N. (1981). Polyurethanes prepared from oxypropylated lignin. Polym. Sci. USSR.

[16] Cateto C.A., Barreiro M.F., Rodrigues A.E., Belgacem M.N. (2009). Optimization Study of Lignin Oxypropylation in View of the Preparation of Polyurethane Rigid Foams. Ind. Eng. Chem. Res..

[17] Perez-Arce J., Centeno-Pedrazo A., Labidi J., Ochoa-Gomez J.R., Garcia-Suarez E.J. (2020). A novel and efficient approach to obtain lignin-based polyols with potential industrial application. Polym. Chem..

[18] Gómez O., Ramón J., Arce P., Madurga J.M., Suárez B.G., José E. (2020). Lignin-Based Polyols.

[19] Bednarek M., Kubisa P. (1998). Copolyethers with controlled structure: Mechanism of formation and microstructure. Macromol. Symp..

[20] Bednarek M., Kubisa P. (1999). Mechanism of cyclics formation in the cationic copolymerization of tetrahydrofuran with ethylene oxide in the presence of diols. Macromol. Chem. Phys..

[21] Bednarek M., Biedron T., Kubisa P., Penczek S. (1991). Activated monomer polymerization of oxiranes. Micro-structure of polymers vs. kinetics and thermodynamics of propagation. Makromol. Chem. Macromol. Symp..

[22] Tokar R., Kubisa P., Penczek S., Dworak A. (1994). Cationic polymerization of glycidol: Coexistence of the activated monomer and active chain end mechanism. Macromolecules.

[23] Auriemma F., Alfonso G.C., De Rosa C. (2017). Polymer Crystallization I.

[24] Kuzayev A.I., Komratov G.N., Korovina G.V., Entelis S.G. (1970). Copolymerization of tetrahydrofuran and epichlorohydrin on boron trifluoride tetrahydrofuranate. Polym. Sci. USSR.

[25] Blanchard L.P., Aghadjan A., Malhotra S.L. (1975). Cationic Copolymerization of Styrene Oxide with Propylene Oxide. J. Macromol. Sci. Part A Chem..

[26] Yamashita Y., Tsuda T., Okada M., Iwatsuki S. (1966). Correlation of cationic copolymerization parameters of cyclic ethers, formals, and esters. J. Polym. Sci. Part A Polym. Chem..

