# Supplementary material for: Lignin-Based Polyols with Controlled Microstructure by Cationic Ring Opening Polymerization
Source: Polymers (Basel). 2021 Feb 22;13(4):651. doi: 10.3390/polym13040651 (PMC7926755; doi:10.3390/polym13040651)
Supplement: Supplementary file 1 [file polymers-13-00651-s001.pdf]

Supporting Information

Lignin-based polyols with controlled microstructure by cationic ring opening polymerization

Jonatan Perez-Arce[1], Ander Centeno-Pedrazo[1], Jalel Labidi[2], Jose R. Ochoa-Gomez,[1,*] Eduardo J. Garcia-Suarez[1,3,4*]

[1]TECNALIA, Basque Research and Technology Alliance (BRTA), Alava Technology Park, Leonardo da Vinci 11, 01510 Vitoria-Gasteiz, Spain.

[2]University of Basque Country UPV/EHU, Department of Chemical and Environmental Engineering, Biorefinery Processes Research Group, Plaza Europa 1, 20018 Donostia-San Sebastián, Spain.

[3]Center for Cooperative Research on Alternative Energies (CIC energiGUNE), Basque Research and Technology Alliance (BRTA), Alava Technology Park, Albert Einstein 48, 01510 Vitoria-Gasteiz, Spain.

[4]IKERBASQUE, Basque Foundation for Science, Maria Diaz de Haro 3, 48013 Bilbao, Spain.

S1. a) TGA profile of the OL, b) first derivative of the TGA of the OL

S2. ATR-FTIR spectra of the LBPs obtained at different BO/OH-L molar ratios.

S3. a) TGA profiles of the LBPs obtained at different BO/OH-L molar ratios; b) first derivatives of the TGA at different BO/OH-L ratios.

S4. ATR-FTIR spectra of the LBPs obtained at different OL concentrations: **a**) BO/OH-L molar ratio 1 and **b**) BO/OH-L molar ratio 2.

S5. a) TGA profiles of the LBPs obtained at different OL concentrations with BO/OH-L molar ratio 1; b) first derivative of the TGA obtained at different OL concentrations with BO/OH-L molar ratio 1; c) TGA profiles of the LBPs obtained at different OL concentrations with BO/OH-L molar ratio 2 and d) first derivative of the TGA obtained at different OL concentrations with BO/OH-L molar ratio 2.

S6. ATR-FTIR spectra of the LBPs obtained at different reaction temperatures.

S7. a) TGA profiles of the LBPs obtained at different reaction temperatures and b) first derivative of the TGA at different reaction temperatures.

S8. ATR-FTIR spectra of the LBPs obtained at different oxirane specific addition flow rates (Q_s).

S9. a) TGA profiles of the LBPs obtained and b) first derivative of the TGA, at different oxirane specific addition flow rates (Q_s).

S10. GPC chromatograms of the obtained LBPs with different oxiranes.

S11. ATR-FTIR spectra of the LBPs obtained with different oxiranes.

S12. a) TGA profiles of the LBPs obtained with different oxiranes and b) first derivative of the TGA obtained with different oxiranes.

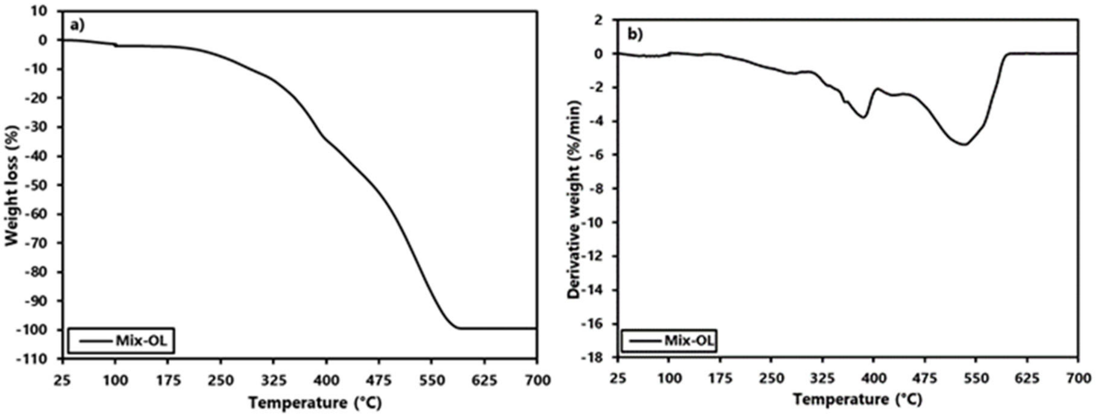

S1. a) TGA profile of the OL, b) first derivative of the TGA of the OL.

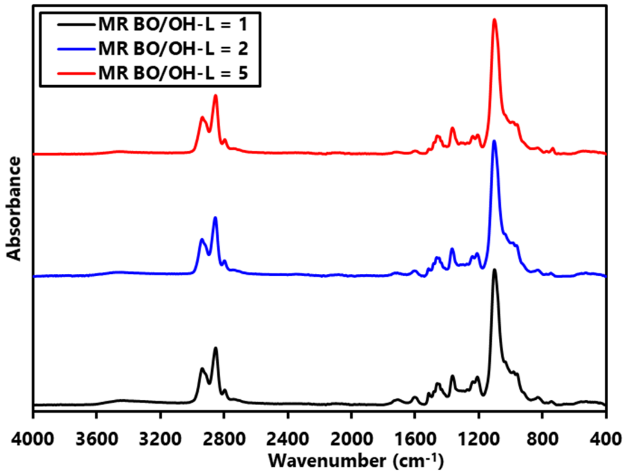

S2. ATR-FTIR spectra of the LBPs obtained at different BO/OH-L molar ratios.

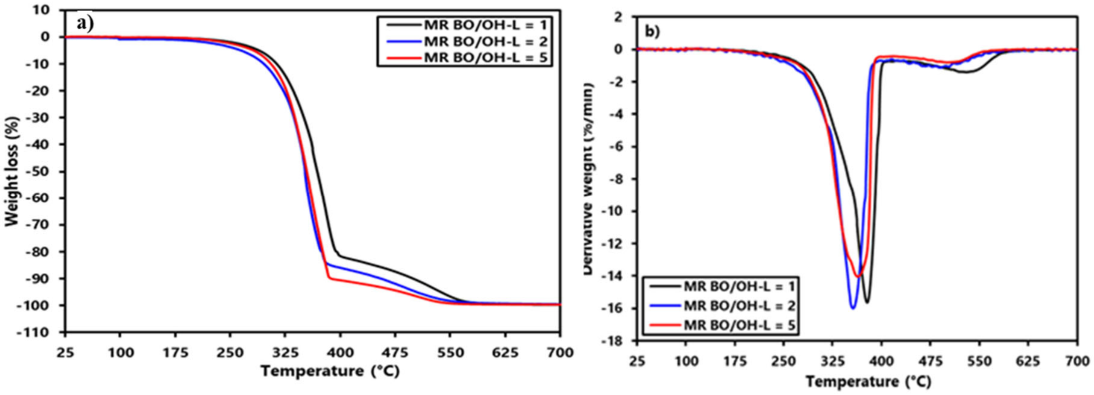

S3. a) TGA profiles of the LBPs obtained at different BO/OH-L molar ratios; b) first derivatives of the TGA at different BO/OH-L ratios.

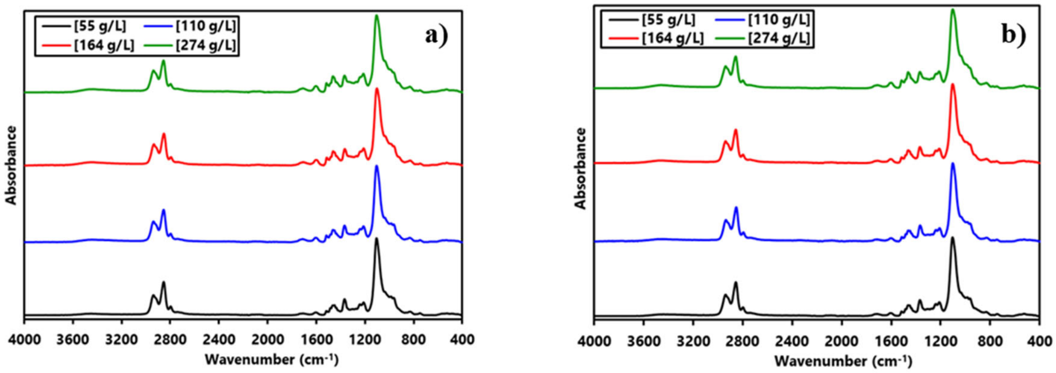

S4. ATR-FTIR spectra of the LBPs obtained at different OL concentrations: **a**) BO/OH-L molar ratio 1 and **b**) BO/OH-L molar ratio 2.

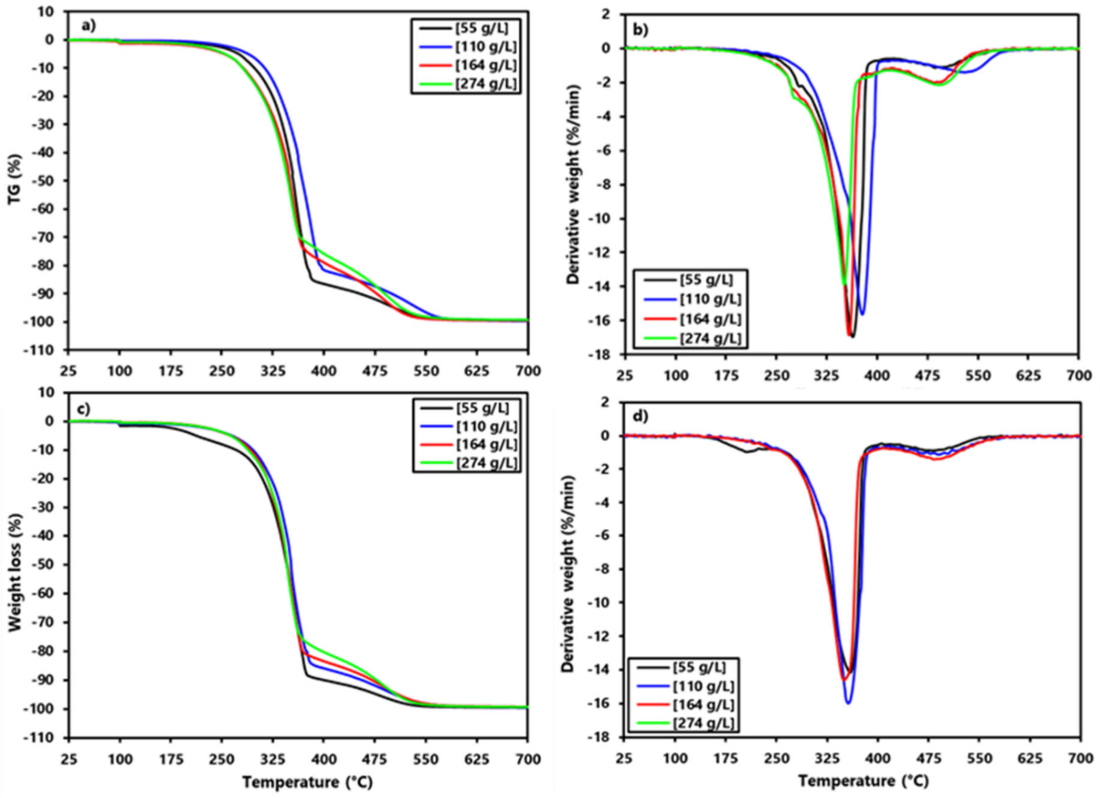

S5. a) TGA profiles of the LBPs obtained at different OL concentrations with BO/OH-L molar ratio 1; b) first derivative of the TGA obtained at different OL concentrations with BO/OH-L molar ratio 1; c) TGA profiles of the LBPs obtained at different OL concentrations with BO/OH-L molar ratio 2 and d) first derivative of the TGA obtained at different OL concentrations with BO/OH-L molar ratio 2.

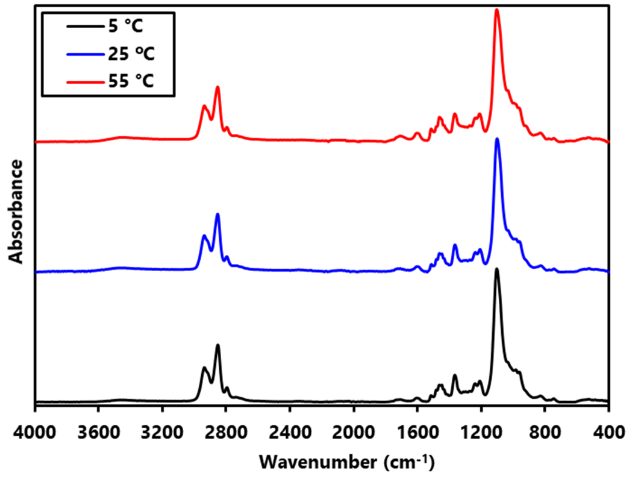

S6. ATR-FTIR spectra of the LBPs obtained at different reaction temperatures.

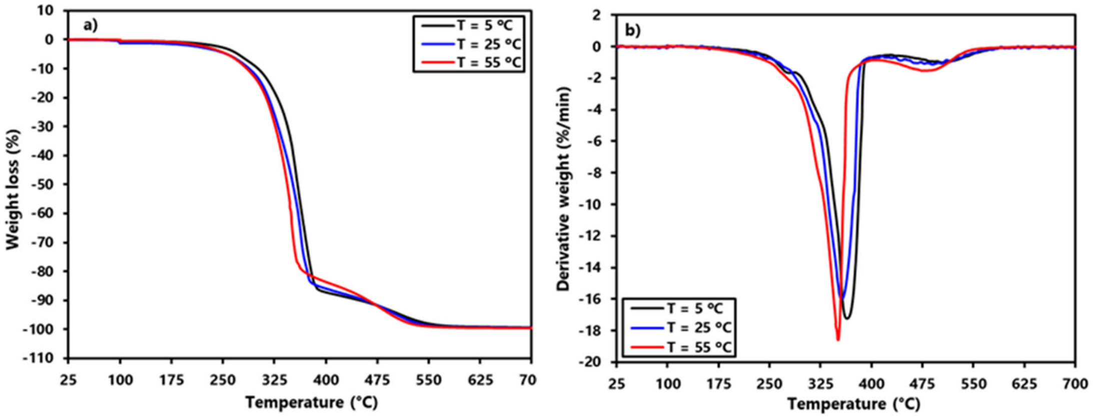

S7. a) TGA profiles of the LBPs obtained at different reaction temperatures and b) first derivative of the TGA at different reaction temperatures.

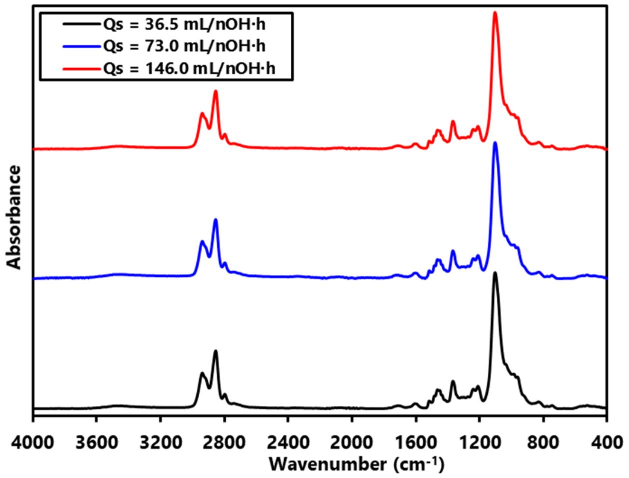

S8. ATR-FTIR spectra of the LBPs obtained at different specific flow rate (Q_s).

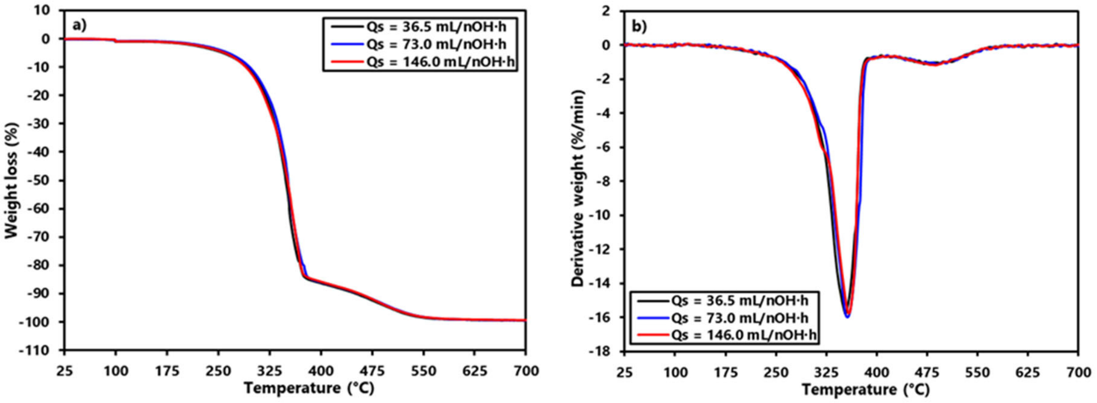

S9. a) TGA profiles of the LBPs obtained at different specific flow rate (Q_s) and b) first derivative of the TGA at different specific flow rate (Qs).

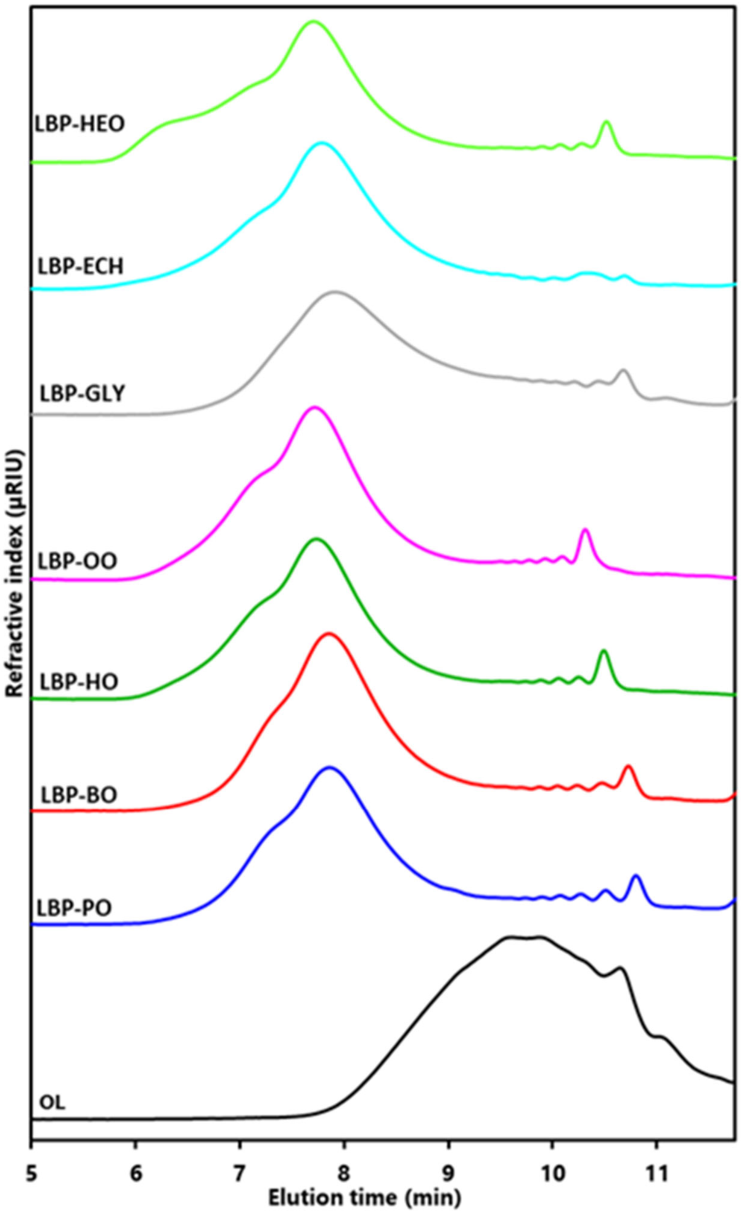

S10. GPC chromatograms of the obtained LBPs with different oxiranes.

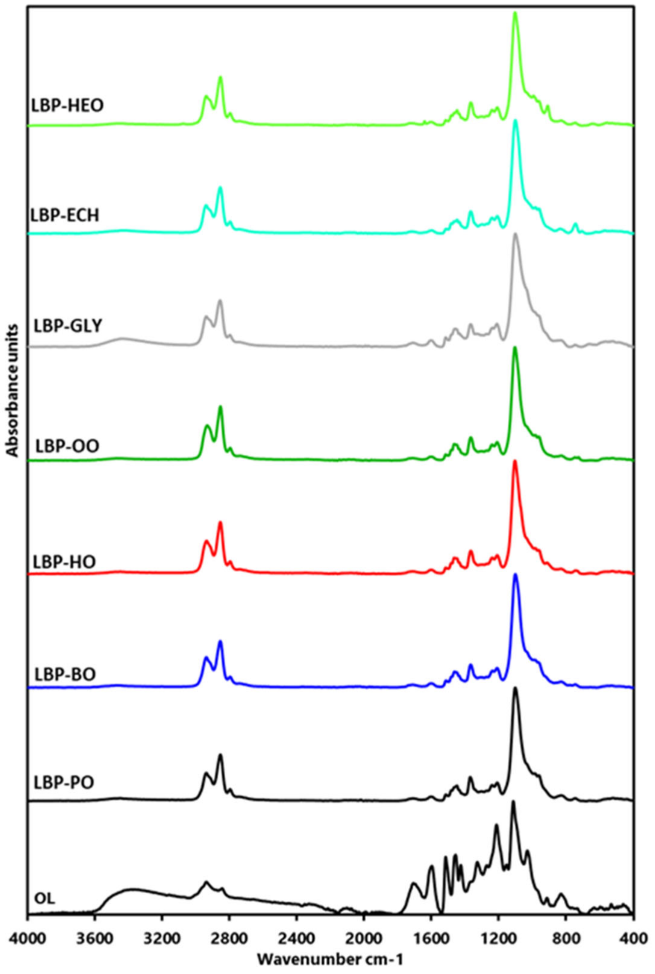

S11. ATR-FTIR spectra of the LBPs obtained with different oxiranes.

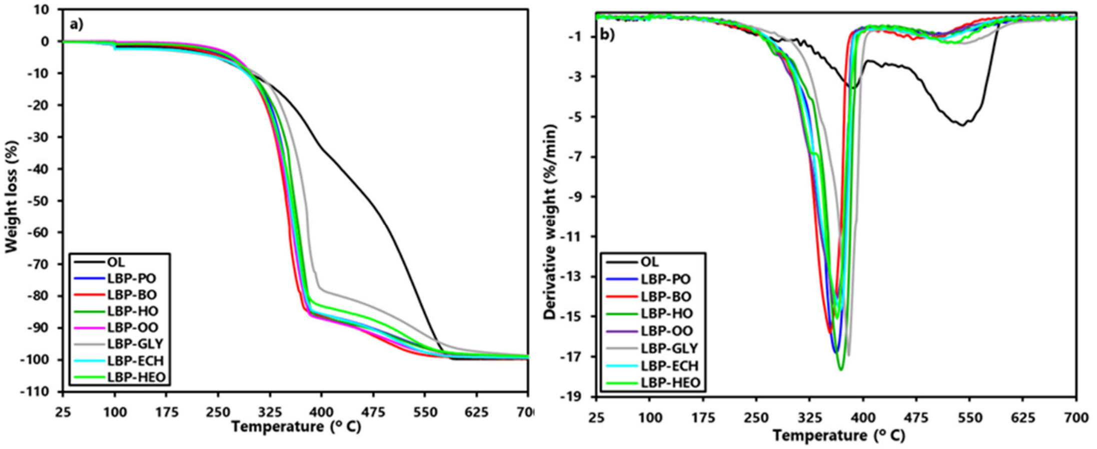

S12. a) TGA profiles of the LBPs obtained with different oxiranes and b) first derivative of the TGA obtained with different oxiranes.
